# Targeted exome sequencing identifies mutational landscape in a cohort of 1500 Chinese patients with non-small cell lung carcinoma (NSCLC)

**DOI:** 10.1186/s40246-021-00320-9

**Published:** 2021-04-12

**Authors:** Ya-jun Zhou, Wei Zheng, Qing-hua Zeng, Yang Ye, Ce Wang, Cheng Fang, Chao-jun Liu, Li Niu, Li-ming Wu

**Affiliations:** 1 Department of Thoracic Surgery, The First Affiliated Hospital of Nanchang University, Nanchang, 330006 Jiangxi China; 2 Department of Oncology, PLA General Hospital, Beijing, 100037 China; 3 Department of Respiratory Medicine, The First Affiliated Hospital of Nanchang University, Nanchang, 330006 Jiangxi China; 4 Department of Cardiothoracic Surgery, Jingdezhen First People’s Hospital, Jingdezhen, 33300 Jiangxi China; 5 CheerLand Clinical Laboratory Co., Ltd., Building 15, Peking University Medical Industrial Park, Zhongguancun Life Science Park, Beijing, 102206 China; 6 Department of Oncology, The 334 Affiliated Hospital of Nanchang University, Nanchang, 330024 Jiangxi China; 7 Department of Radiation Oncology, The First Affiliated Hospital of Anhui Medical University, No.218, Jixi Road, Hefei, 230022 Anhui China

**Keywords:** Targeted exome sequencing, Non-small cell lung carcinoma, Chinese patients, Cancer, Disease

## Abstract

**Background:**

Non-small cell lung carcinoma (NSCLC) is one of the most common human cancers, comprising approximately 80–85% of all lung carcinomas. An estimated incidence of NSCLC is approximately 2 million new cases per year worldwide.

**Results:**

In recent decade, the treatment of NSCLC has made breakthrough progress owing to a large number of targeted therapies which were approved for clinical use. Epidemiology, genetic susceptibility, and molecular profiles in patients are likely to play an important factor in response rates and survival benefits to these targeted treatments and thus warrant further investigation on ethnic differences in NSCLC. In this study, a total number of 1500 Chinese patient samples,1000 formalin fixed paraffin-embedded (FFPE) and 500 blood samples, from patients with NSCLC were analyzed by targeted sequencing to explore mutational landscape in ethnic groups associated with China.

**Conclusions:**

Overall, the data presented here provide a comprehensive analysis of NSCLC mutational landscape in Chinese patients and findings are discussed in the context of similar studies on different ethnic groups.

## Background

Non-small cell lung cancer (NSCLC) represents a heterogeneous group of lung cancer. Two major NSCLC subtypes are distinguished: the adenocarcinoma (AD) and the squamous cell carcinoma (SCC). In general, treatments for NSCLC can include chemotherapy, targeted drug therapy, immunotherapy, surgery, and palliative procedures. Ideal treatment options depend on whether the cancer has already spread and metastasized, what are the genetic changes in the cancer cells, and the patients’ overall health and age. Sequencing of tumor sample may help to screen the patients who may response to and benefit from targeted treatments and help to lower the mortality rate [1]. For instance, if one of the previously identified NSCLC-associated genes, such as *EGFR*, *ALK*, *ROS1*, *BRAF*, *RET*, *MET*, or *NTRK*, is mutated in the patient’s cancer cells, targeted therapies has to be considered [1]. Accordingly, the National Comprehensive Cancer Network (NCCN) NSCLC guidelines had recommended the routine detection of EGFR or ERBB2 mutations, or ALK, ROS1, or RET fusions prior to treatment. However, previous studies raised the possibility that the distribution of these mutations show a race-dependent pattern, with one study estimating that 10% of Caucasians but as high as 50% of Asians will be found to have drug sensitizing mutations of the EGFR [2]. The observed high variation in mutation frequency in demographic subgroups urges for large-scale studies that systematically investigate mutation landscapes in certain races and offers a better insight what genes has to be tested prior to choosing a targeted therapy [3, 4].

Next-generation sequencing (NGS) has revolutionized the identification process and systematic characterization of genomic alterations, including single nucleotide variations and small insertions/deletions (InDels), and will likely receive recommendations from cancer societies in the very near future about its daily use in clinical oncology practice. Indeed, upfront tumor genotyping is now widely considered as an essential step in guiding treatment decision-making in the management of patients with NSCLC [5].

In this study, a number of 1000 formalin-fixed paraffin-embedded (FFPE) and 500 blood samples with NSCLC were analyzed by NGS-targeted sequencing. This study represents to our knowledge one of the largest efforts so far to systematically characterize mutational landscape in Chinese NSCLC cohort samples.

## Results

### Clinical features of the patient samples

Discovery and quantification of genetic alterations in NSCLC, from point mutations to large genomic rearrangements, requires a comprehensive genome-wide approach and a large sample cohort. We have collected 1000 formalin-fixed paraffin-embedded (FFPE) tumor samples and 500 blood samples from a total of 1500 patients diagnosed with NSCLC between June 2017 and April 2019. Tissue and blood samples were obtained from independent patient groups. The detailed clinical characteristics of the patients are shown in Table 1. Briefly, lung adenocarcinoma accounted for 84.3% of the FFPE samples (843/1000), squamous cell carcinoma for 14.2% (142/1000), and others for 1.5% (15/1000). As for the blood samples, lung adenocarcinoma accounted for 80.4% (402/500), squamous cell carcinoma for 17% (85/500), and others for 2.6% (13/500). In total, 39 samples were excluded due to not passing quality standards along the sample processing and sequencing.

**Table 1:** Overview of patient and tumor characteristics in the present study

Characteristics	Tissues (1000 cases) *n* (%)	Blood (500 cases) *n* (%)
Sex		
Male	578 (57.8)	308 (61.6)
Female	422 (42.2)	192 (38.4))
Age		
>60	392 (39.2)	164 (32.8)
≤60	602 (60.2)	334 (66.8)
Unknown	6 (0.6)	2 (0.4)
Smoking		
Non-smoking	945 (94.5)	477 (95.4)
Occasionally	11 (1.1)	3 (0.6)
Often	44 (4.4)	20 (4)
Tumor type		
Lung adenocarcinoma	843 (84.3)	402 (80.4)
Lung squamous carcinoma	142 (14.2)	85 (17)
Unknown	15 (1.5)	13 (2.6)

### Overview of the genomic alterations of 1000 tissue and 500 blood samples of NSCLC patients

**Fig. 1:**
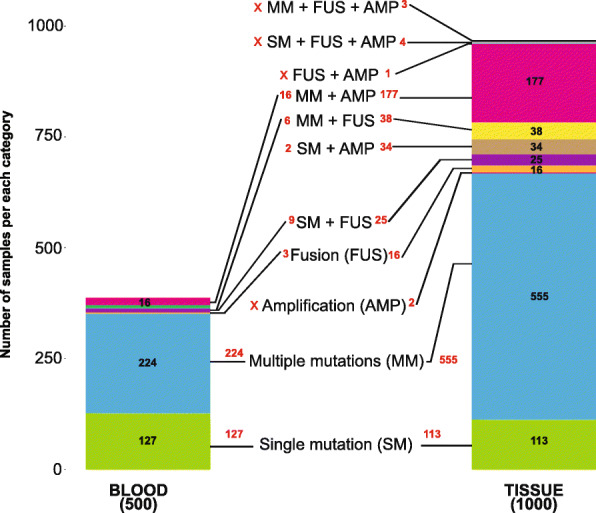
Overview of the genomic alterations of 1000 tissue and 500 blood samples of NSCLC patients. Distribution of tissue and blood samples with single mutation (single base variation, insertion or deletion, SM); multiple single mutations (MM); amplification (AMP), fusion (FUS) or combination of these

The clinical significance of identifying hypermutated tumors has recently been demonstrated in several NSCLC studies [6, 7]. However, there is a large variability in mutation burden within tumor types in NSCLCs [8]. To begin to explore the mutation burden in our cohort, we first identified the overall mutation landscape across the tissue and blood samples. We subclassified mutations into four main types, single mutation (single base variation, insertion or deletion, SM), multiple single mutations (MM), amplification (AMP), and fusion (FUS) (Fig. 1). As for the FFPE NSCLC tissue samples, a total of 968/1000 samples had at least one type of the above-listed mutations, while 387/500 blood NSCLC samples were found to belong to one of the mutation groups. Specifically, there were 127/500 (25.4%) blood samples with single base variation, 224/500 (44.8%) with multiple mutations. Only 36/500 (7.2%) blood samples showed amplification or fusion (Fig. 1). As for tissue samples, there were 113/1000 (11.3%) single base variation, 555/1000 (55.5%) with multiple mutations, and 221/1000 (22.1%) samples had amplification alone or in combination with other mutations. In contrast to 117/500 (22.6%) of blood samples, only 32/1000 tissue samples (3.2%) had not detected mutation within the studied 65 genomic regions (Fig. 1).

### Mutation patterns of frequently altered cancer genes

**Fig. 2:**
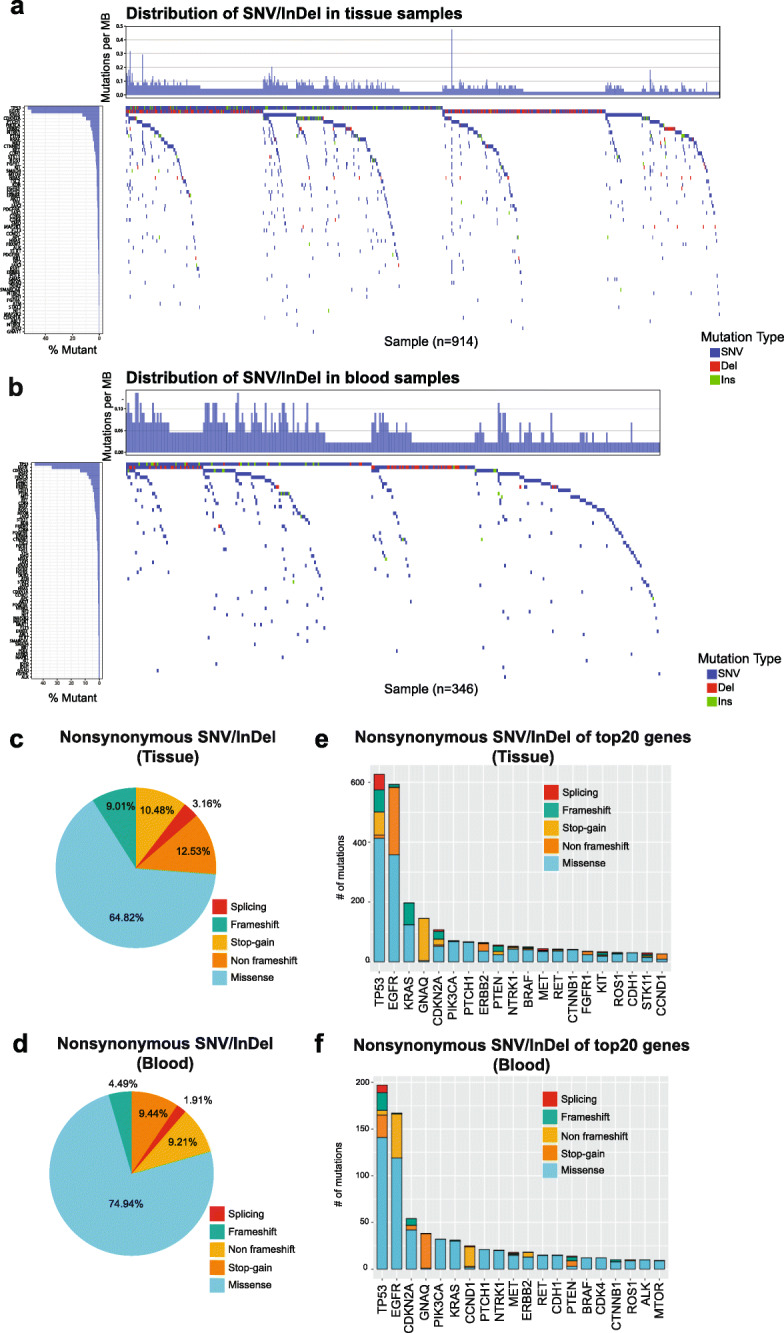
Significantly mutated genes in NSCLC. Waterfall plot of the distribution of SNV/InDel mutations found in tissue (**a**) and blood (**b**) patient samples. The top plot show number of mutations per Mb sequenced for a cohort of 914 NSCLC samples. Left plot shows the frequency of samples mutated for the listed gene. The central plot shows the types of mutations (SNV, Insertion, Deletion) in each sample. The distribution of nonsynonymous frameshift insertions and deletions, missense mutations, Stop-gain, and other infrequent alterations (e.g. splicing) in both the tissue (**c**, **e**) and blood samples (**d**, **f**)

Next, we set out to determine the most common cancer genes enriched for SNV/InDel in our NSCLC patient cohort. We identified many genes previously also found to be mutated in NSCLC, including several tumor suppressor genes *TP53* [9], *CDKN2A* [10], and oncogenes *EGFR* [11] and *KRAS* [12]. Notably, we observed highly accumulated TP53 and EGFR mutations in both blood and tissue samples of NSCLC patients (Fig. 2a, b). Co-occurrence of EGFR with the TP53 mutations was remarkable in the tissue samples (>25%). EGFR mutation rate was significantly higher in tissues (~55%) vs. blood (~35%). In addition, we found several other genes that were significantly mutated in our cohort, such as PTCH1 and PIK3CA (Fig. 2a, b). Other, less frequently detected, but previously identified genes included tumor suppressor genes (*APC*) and tyrosine kinase genes (*ERBB2*, *FGFR*, and *NTRK* genes).

Next, we assessed the distribution of nonsynonymous frameshift insertions and deletions, missense mutations, Stop-gain, and other infrequent alterations (e.g., splicing) in both the tissue and blood samples (Fig. 2c–e). In addition to identifying previously known NSCLC-associated genes, such as *TP53*, *KRAS*, *EGFR*, and *CDKN2A*, the analysis revealed GNAQ gene, which was previously mostly implicated in melanomas and only a very recent study linked to lung cancer (Fig. 2c–e) [13]. Identified mutations of GNAQ included p.R60G, p.P174R, p.A93D, p.M59L, and p.Q81H.

### Recurrent SNV mutations in NSCLC

**Fig. 3:**
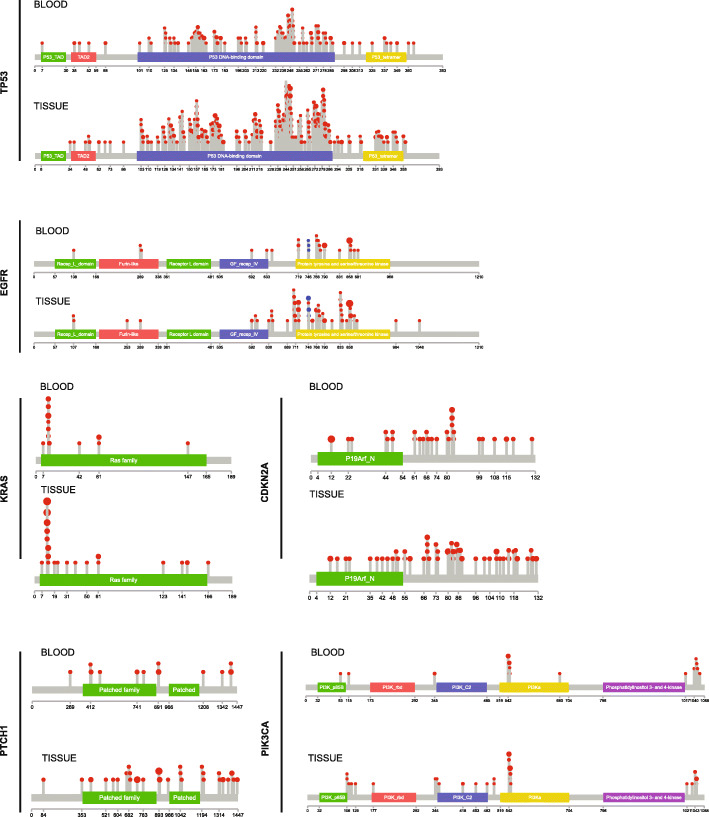
Recurrent SNV mutations in TP53, EGFR, KRAS, CDKN2A, PTCH1, and PIK3CA. Positional distribution of SNV mutations across blood and tissue NSCLC samples. SNV mutations detected by exome sequencing are depicted on lolliplot and mapped to the structure of the corresponding gene

Next, we explored the positional distribution and recurrence of SNV mutations in the genes with most frequent mutations, focusing on the most frequently mutated genes, TP53, EGFR, KRAS, CDKN2A, PTCH1, and PIK3CA (Fig. 3).

Most clinical studies suggest that lung cancer with alterations detected in TP53 carries an overall worse prognosis and such cases are more resistant to chemotherapy and radiation [14]. Indeed, as it was shown in Fig. 2, mutations of the TP53 gene occurred in over 50% of NSCLC samples in our cohort. In our cohort, only 8 samples showed mutations at codons 157, 6 samples at codon 158, 11 samples at codon 179, and 27 samples at codon 248 of TP53. These codons are typically mutated in lung cancer from smokers and uncommonly observed in lung cancer from nonsmokers [15].

Previous analysis of the TK domain of the EGFR by Shigematsu et al*.* identified that all mutations in lung cancer specimens occurred within exons 18–21, with a prevalence of 21% [11, 16, 17]. Consistent with these previous reports, EGFR mainly had three subtype of mutation (p.L858R, Exon 19del, p.T790M). EGFR p.L858R and Exon 19del were the most common EGFR active mutant, which may be sensitive to EGFR-TKI inhibitors such as gefitinib, erlotinib, or afatinib. We found the percentage of these mutation in FFPE and blood sample were similar. There were 42.4% p.L858R in blood sample and 44.4% in FFPE samples. Similarly, there were 38.5.4% Exon 19del in blood sample and 34.2% in FFPE samples. Interestingly, there was significantly different percent of p.T790M in FFPE and blood sample. The percent of p.T790M in FFPE and blood sample were 24.8% and 2.4%, respectively.

We found that mutations in KRAS were mostly detected at amino acid positions 12, 13, 61, in regions which are considered mutational hotspots (Fig. 3). Recurrent mutations included p.G12C, p.G12V, p.G13D, and p.Q61H. In addition, we have also found pA146T in two tissue samples.

In addition to the previously described mutations involving *TP53*, *EGFR*, and *KRAS* genes, our analysis in this large cohort revealed several other recurrent point mutations in NSCLC. For instance, recurrent point mutations (E545K) in the *PIK3CA* gene were identified. In fact, somatic mutations of the *PIK3CA* gene have been also described NSCLC [18, 19].

CDKN2A gene mutation was detected in ~10% of the analyzed NSCLC tissue samples. CDKN2A is a well-known tumor suppressor, which regulates cell cycle progression by inhibiting cyclinD-CDK4 and cyclinD-CDK6 complexes responsible for initiating the G1/S phase transition. Recurrent mutations included p.A68V, p.R80X, p.A85P, p.D108Y, p.E120X, and p.V115E.

Recently, the *PTCH1* gene mutations were also identified in NSCLC. Previous studies found that the most common genetic alterations in PTCH1 are missense mutations (2.17%), frameshift (0.46%), nonsense mutations (0.17%), and S1203Afs*52 (0.15%) [20]. We found p.A741V, p.D898N recurrent mutations (Fig. 3).

### Structural rearrangement signatures and overview of aberration frequencies identified in our NSCLC patient cohort

**Fig. 4:**
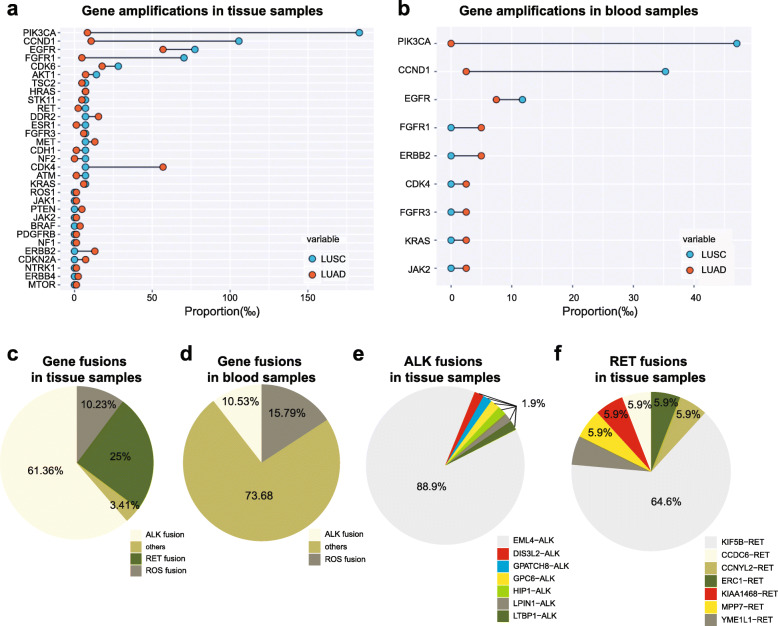
Amplifications and gene fusion signatures identified in our NSCLC patient cohort. Structural rearrangement signatures identified in Chinese NSCLC patients. Rearrangement hotspots identified in NSCLC patients. (**a**) Gene amplificaitons in tissue samples; (**b**) Gene amplificaitons in blood samples; (**c**) Gene fusions in tissue samples; (**d**) Gene fusions in blood samples; (**e**) ALK fusions in tissue samples; (**f**) RET fusions in tissue samples

Previous studies have been able to detect significant copy number alteration in lung adenocarcinomas [21, 22]. Sequencing of the coding exons of the 65 pre-selected candidate cancer genes in our study identified gene amplifications in both lung adenocarcinoma (LUAD) and lung squamous cell carcinoma (LUSC) (Fig. 4a, b). Similarly to previous reports, we have found both *EGFR* and *KRAS* gene copy number gains to occur frequently in NSCLC [23, 24].

The recent discovery of a fusion involving the echinoderm microtubule-associated protein-like 4 (EML4) and anaplastic lymphoma kinase (*ALK*) genes in tumor specimens from a subset of patients with NSCLC (mostly adenocarcinoma) and the quite effective treatment of these cases by ALK kinase inhibitors have reinvigorated efforts to identify additional genomic rearrangements that could be therapeutic targets [8, 25]. Thus, we also analyzed the tumor genomes for fusion genes and were able to systematically identify fusion genes (Fig. 4c). ALK fusion mutation was very common in our NSCLC cohort. We found that among the samples which had any type of genomic rearrangements, ~61% FFPE and ~74% blood samples had rearrangements related to ALK. The most common rearrangement of ALK in tissue samples was EML4-ALK (88.9%), and the other subtypes included GPC6-ALK (1.9%), LTBP1-ALK (1.9%), GPATCH8-ALK (1.9%), DIS3L2-ALK (1.9%), HIP1-ALK (1.9%), and LPIN1-ALK (1.9%) (Fig. 4e). The most common rearrangement of RET in tissue samples was KIF5B-RET (64.7.9%), and the other subtypes included MPP7-RET (5.8%), CCNYL2-RET (5.8%), KIAA1468-RET (5.8%), CCDC-RET (5.8%), and YME1L1-RET (5.8%) (Fig. 4e).

### Combination of SNV, amplification, and fusion of significantly mutated genes

**Fig. 5:**
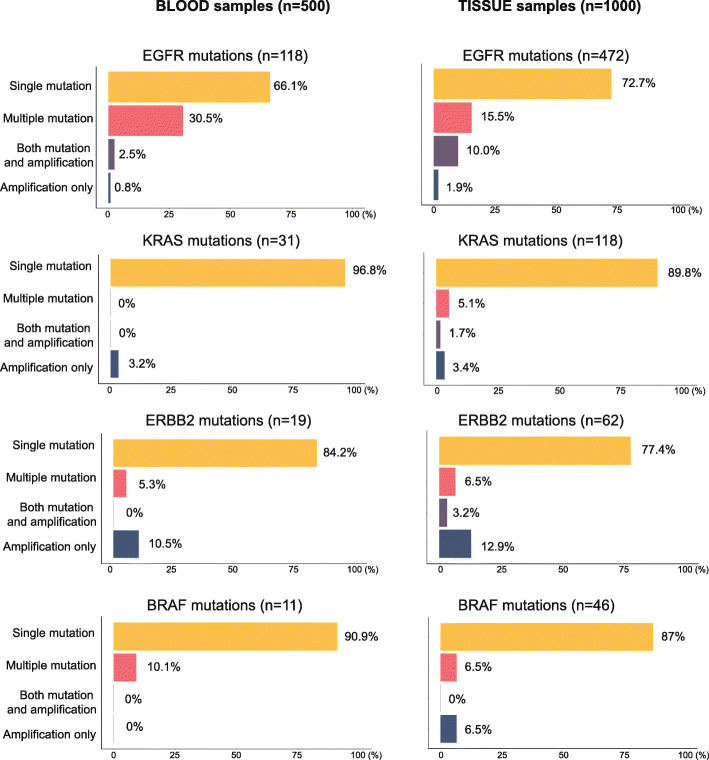
An overview of significantly mutated genes. Assessment of single mutations (SNVs and InDels), multiple mutations, and amplifications across the top most frequently mutated genes, excluding TP53. Genes were depicted according to aberration frequencies

Finally, to further explore the mutations in the most common cancer genes involved in Chinese NSCLC patients, we also assessed the co-occurrence of single nucleotide variations with other mutational events. Strikingly, majority of samples (~90%) carrying KRAS mutations were not containing any other type of mutations (Fig. 5). In contrast, EGFR has often co-occurred with other mutations.

## Discussion

In this study, we analyzed genomic events in a large set of FFPE and blood samples from patients with NSCLC. Specifically, we used targeted sequencing of selected candidate genes to identify most common mutations in a large cohort of Chinese NSCLC patients. The vast amount of genomic information generated in this and similar studies is expected to transform our current understanding of lung cancer and advance personalized lung cancer therapy. We also anticipate that our study along with other studies implementing tumor mutation landscape analysis using targeted and genome-wide NGS across different ethnic groups in lung cancer will enormously expand our knowledge base in lung cancer biology, treatment strategy, new drug target development, and NSCLC outcome.

In fact, recent discoveries made based on previous mutational analysis already significantly improved and expanded the availability of targeted therapies. Development of new receptor kinase inhibitors, such as erlotinib and gefitinib (against EGFR) and most recently crizotinib (against rearranged ALK), and antibodies such as cetuximab (against EGFR) are all great examples how NGS can help to improve personalized medicine [26]. However, while these drugs are effective in a subset of patients, our analysis and other studies clearly suggest a very complex mutational landscape in NSCLC and warrant for even more targeted drug development to be able to further decrease the still high mortality rate of NSCLC.

An interesting target that came out from our analysis is GNAQ (Fig. 2). GNAQ (guanine nucleotide binding protein [G protein], q polypeptide) is known as a subunit of one of the heterotrimeric guanine nucleotide binding proteins (G proteins) that is involved in multiple processes of mammary cells including hormonal signal transduction, metabolism, development, cell survival, and sensory functions. Previous studies mostly implicated its mutations in melanoma, and GNAQ mutations have not been documented in NSCLC. We found several nonsynonymous SNV (Stop-gain) in GNAQ both in blood and tissue samples, though none of the identified mutations were shown recurrence across the samples.

Another interesting candidate for follow-up studies was the tumor suppressor Patched 1 (PTCH1), a multi-pass transmembrane protein which is over-expressed in many metastatic cancers. In an unbound inactive state, PTCH1 acts as a negative regulator of smoothened (SMO), while upon activation it leads to activation of GLI1 proto-oncoprotein. Since PTCH1 is a multidrug transporter, it contributes to chemotherapy resistance by the efflux of chemotherapeutic agents such as doxorubicin [27]. PTCH1-altered tumors can be now targeted with three different FDA-approved SMO inhibitors, namely sonidegib, vismodegib, and glasdegib [27].

An important context to discuss is related to health disparities, which are a recognized and well-documented phenomenon on the cancer field but has not yet been addressed in case of NSCLC. Socioeconomic and cultural differences across ethnic groups undoubtedly account for some of the disparities, namely that certain groups may bear a disproportionate burden of cancer compared with other groups. Our study specifically aimed to collect and explore data of a well-defined group of patients based on geographic location. Our data collection and/or exploration did not yet include gathering information on income, education, disabilities, and other possibly relevant characteristics. Nevertheless, it is important to highlight that the analyzed samples are all representing non-smoker patients and we gathered information on gender that will be further correlated with mutational landscapes in follow-up studies.

While a number of cancer centers have already begun to integrate molecular profiling and even clinical next-generation sequencing (NGS) into the pipeline of routine cancer diagnosis in order to increase accuracy and efficiency of treatments, it is important to recognize and discuss the limitations of the targeted therapy in the treatment of NSCLC. For instance, EGFR inhibitors, such as gefitinib, erlotinib, or afatinib, can effectively shrink tumors for several months; these drugs eventually stop working for most patients, usually because the cancer cells within the tumor develop additional mutation(s) in the *EGFR* gene. Studies investigating the clinicopathological factors influencing post-recurrence survival and the effect of post-recurrence therapy in NSCLC will be critical to further advance therapies.

## Conclusions

In summary, using targeted whole exome sequencing, we have identified mutations in a large cohort of Chinese NSCLC blood and tissue samples for 65 genes and provide an overview of the mutational landscape by analyzing CNVs, fusions, and SNV/InDel in details.

## Methods

### Samples

The study was conducted in accordance with the Helsinki Declaration and was approved by the institute’s Ethics Committee. All the patients enrolled had been informed about the content and purposes of this study and signed the consents. In this study, we have collected and processed a total of 1000 formalin-fixed paraffin-embedded (FFPE) tumor samples and 500 blood samples of patients diagnosed with NSCLC between June 2017 and April 2019. Patient samples were collected from The First Affiliated Hospital of Nanchang University (Nanchang, Jiangxi, China), PLA General Hospital (Beijing, China), Jingdezhen First People’s Hospital (Jingdezhen, Jiangxi, China), 334 Affiliated Hospital of Nanchang University (Nanchang, Jiangxi, China), and The First Affiliated Hospital of Anhui Medical University (Hefei, Anhui, China). Tissue and blood samples were collected from independent patient groups.

### DNA extraction and Next-Generation Sequencing

Genomic profiling was performed in a College of American Pathologists (CAP)-accredited lab at OrigiMed (Shanghai, China) according to standard procedures. Briefly, genomic DNA was extracted from tissue and plasma samples were tested for cell-free DNA (cfDNA). DNA was extracted from tissue and liquid blood biopsies using standard DNA Extraction Kit (QIAamp DNA FFPE Tissue Kit; Qiagen, Hilden, Germany) and MagMAX Cell-free DNA isolation kit (Thermo, Cat#A29319), respectively, according to manufacturer’s recommendations. A total of 3.6–35 ng of DNA was used as input to prepare barcoded libraries for each sample. The exon regions of 65 cancer driver genes were tested using the IDT (Integrated DNA Technologies, Coralville, IA, USA) custom-designed panel. The genes included in this panel are ABL1, AKT1, ALK, APC, AR, ATM, BRAF, CCND1, CDK4, CDK6, CDKN1A, CDKN2A, CTNNB1, DDR2, EGFR, ERBB2, ERBB3, ERBB4, ESR1, FBXW7, FGFR1, FGFR2, FGFR3, FGFR4, FLT3, GNA11, GNAQ, GNAS, HRAS, IDH1, IDH2, JAK1, JAK2, JAK3, KDR, KIT, KRAS, MEK1, MET, MTOR, NF1, NF2, NRAS, NTRK1, NTRK2, NTRK3, PDGFRA, PDGFRB, PIK3CA, POLE, PTCH1, PTEN, RB1, RET, ROS1, SATA3, SMAD4, SMARCA4, SMO, STK11, TERT, TP53, TSC1, TSC2, and VHL.

The FFPE and blood samples were sequenced by Illumina Nova seq. As for the FFPE samples, the mean sequencing depth was nearly 1200x, the coverage rate was 99.99%, and fraction of bases mapped to target region was between 40 and 70%. At least 200x nucleic acid coverage and 1% of mutation allele fraction were used as the standard cutoff to make the final variant call. As for the blood samples, the mean sequencing depth was nearly 10000x, the coverage rate was 99.99%, and fraction of bases mapped to target region was between 4 and 70%. At least 2000x nucleic acid coverage and 0.5% of mutation allele fraction were used as the cutoff for the final variant call.

### Bioinformatics analysis

Our initial analysis aimed to explore genomic alterations, including gene rearrangements, copy number variations (CNVs), single nucleotide variants (SNVs), and short and long insertions/deletions (InDels). Raw sequencing reads were aligned to the human reference genome (hg19) using Burrows-Wheeler Aligner (BWA). Consensus reads were generated for error suppressing and PCR duplicates were removed using in-house software ECR. Read depth and coverage of the targeted regions were calculated by in-house software LibraryQC. The log-ratio per region of each target genes was calculated, and customized algorithms were used to detect copy number variations. Focal amplifications were characterized as genes with thresholds ≥4 copies. Gene rearrangements and long indels were detected using CREST [28] and Manta [29]. SNVs and short indels were identified by MuTect [30] and Pindel [31].

## Data Availability

All data generated or analyzed during this study are included in this published article. The sequence data will be provided upon request.

## Abbreviations

NSCLC: Non-small cell lung carcinoma
FFPE: Formalin-fixed paraffin-embedded
AD: Adenocarcinoma
SCC: Squamous cell carcinoma
NCCN: National Comprehensive Cancer Network
NGS: Next-generation sequencing
